# Helical versus static approaches to delivering tomotherapy to the junctional target for patients taller than 135 cm undergoing total body irradiation

**DOI:** 10.1186/s40001-022-00886-7

**Published:** 2022-11-24

**Authors:** Mümtaz Köksal, Jonathan Baumert, Felix Schoroth, Thomas Müdder, Davide Scafa, David Koch, Christina Leitzen, Gustavo R. Sarria, Leonard C. Schmeel, Frank A. Giordano

**Affiliations:** 1 Department of Radiation Oncology, University Medical Center Bonn, Bonn, Germany; 2 Department of Radiation Oncology, University Medical Center Mannheim, Mannheim, Germany

**Keywords:** Radiotherapy, Helical tomotherapy, Static tomotherapy, Total body irradiation, Bone-marrow transplantation, Field junction, Overlap region

## Abstract

**Background:**

Helical TomoTherapy^®^ is widely used for total body irradiation as a component of conditioning regimens before allogeneic bone-marrow transplantation. However, this technique limits the maximum length of a planning target volume to 135 cm. Therefore, patients taller than 135 cm require two planning computed tomography scans and treatment plans. The junctional target between these two treatment plans is thus a critical region for treatment planning and delivery. Here, we compare radiation coverage of the junctional target between helical and static approaches to treatment planning and delivery to determine which approach allows high quality irradiation planning and provides more robustness against patient movement.

**Methods:**

We retrospectively analyzed 10 patients who underwent total body irradiation using a static four-field box planning approach and nine patients who underwent total body irradiation using a helical planning approach. All patients were taller than 135 cm. The junctional target volume was divided into 10 slices of 1 cm thickness (JT_1_–JT_10_) for analysis. Dosimetric parameters and dose-volume histograms were compared to assess the quality of coverage of the junctional target between the helical and static planning approaches.

**Results:**

The D_50_ for the total junctional target was slightly higher than the prescribed dose for both helical and static approaches, with a mean of 108.12% for the helical group and 107.81% for the static group. The mean D_95_ was 98.44% ± 4.19% for the helical group and 96.20% ± 4.59% for the static group. The mean homogeneity index covering the entire junctional target volume was 1.20 ± 0.04 for the helical group and 1.21 ± 0.05 for the static group. The mean homogeneity index ranged from 1.08 ± 0.01 in JT_1_ to 1.22 ± 0.06 in JT_6_ for the helical group and from 1.06 ± 0.02 in JT_1_ to 1.19 ± 0.05 in JT_6_ for the static group. There were no significant differences in parameters between helical and static groups. However, the static approach provided robustness against up to 30 mm of lateral movement of the patient.

**Conclusions:**

As long as TBI using helical TomoTherapy^®^ is limited to a maximum length of 135 cm, the junctional target must be addressed during treatment planning. Our analysis shows that the static four-field box approach is viable and offers higher robustness against lateral movement of the patient than the helical approach.

## Background

Total body irradiation (TBI) is an important component of allogeneic bone-marrow transplantation (BMT) conditioning regimens and is used as a myeloablative treatment [1, 2]. Several studies show that TBI is an outcome-improving tool for many diseases, such as acute lymphoblastic leukaemia (ALL), acute myeloblastic leukaemia (AML), and natural killer cell lymphoma [3–5]. One method of delivering TBI before BMT is helical tomotherapy. Not only does helical tomotherapy simplify the process of TBI, it also ensures minimal variance between planned and delivered doses and provides a homogeneous dose distribution [6, 7]. Indeed, several clinical studies provide examples of helical tomotherapy use and demonstrate its feasibility in BMT regimens [8–11].

However, TBI using helical tomotherapy is limited by a maximum treatment length of 135 cm. As a result, patients taller than 135 cm require two planning computed tomography (CT) scans to fully cover the body. These planning CT scans are performed in a cranial-to-caudal direction for the upper body and caudal-to-cranial direction for the lower body, which creates an overlapping junctional volume around the upper thigh region. This overlap presents a challenge to dose planning and delivery in terms of potential over- or underdosage. Furthermore, there is a risk of dose deviation in the junction area, as the position of patients—particularly their legs—changes during the rotation between upper treatment plan delivery and lower treatment plan delivery despite patient fixation. This can result in reduced compatibility between planned and delivered doses, resulting in reduced homogeneity in the junctional area and subtherapeutic doses.

One way to plan junctional target (JT) volumes for TBI for patients taller than 135 cm is by standardized treatment planning using a helical approach, in which irradiation is delivered with gantry rotation and couch translation into the bore, while the multileaf collimator is adjusted as planned throughout treatment if needed. Indeed, previous studies utilizing a helical approach have sought to determine the CT parameters that achieve optimal dose distribution in the JT target when two overlapping scans are required [12–16]. However, an alternative way to plan JT volumes for patients taller than 135 cm is via standardized treatment planning using a static approach with a four-field box. In this manner, no helical movement of the gantry needed, and a fixed jaw setting of the multileaf collimator may be used. This static approach could potentially allow a substantial increase in dose homogeneity against lateral movement during treatment and rotation compared with the helical approach.

To determine whether the static treatment planning approach is feasible, meets International Commission on Radiation Units and Measurements (ICRU) guidelines [2, 17], and achieves dose homogeneity in the JT volume comparable to that of the helical treatment planning approach, we directly compared static versus helical approaches by performing retrospective dosimetric evaluation of 19 patients taller than 135 cm who underwent helical or static planning for TBI.

## Methods

This analysis was conducted for routine quality assurance in line with requirements of the German radiation protection law. Therefore, ethical approval was not required.

### Patients

Data from all patients taller than 135 cm at the time of TBI delivery between 2012 and 2020 who underwent planning with a direct field connection between upper and lower CT scans were analyzed. Patients who underwent planning using a dose gradient in the JT volume were excluded.

### Treatment planning

In preparation for TBI, two planning CT scans were required, because the TomoTherapy® Hi-ART II is limited to a maximal couch movement of 135 cm. These CT scans were performed using a fixation mask for the head and a vacuum cushion for the body to help stabilize the patient and prevent significant alterations in the patient’s position during CT, between CT and treatment, and during treatment. The first CT scan was performed in the cranio-to-caudal direction, and the second CT scan was performed in the caudo-to-cranial direction, both with a slice thickness of 5 mm. To correctly match these two scans and assist in treatment planning, a radio-opaque marker was placed on the patient’s upper thigh. The exact position of the marker depended on the patient’s height to ensure that neither CT scan (and thus treatment plan) exceeded the maximum length of 135 cm.

**Fig. 1:**
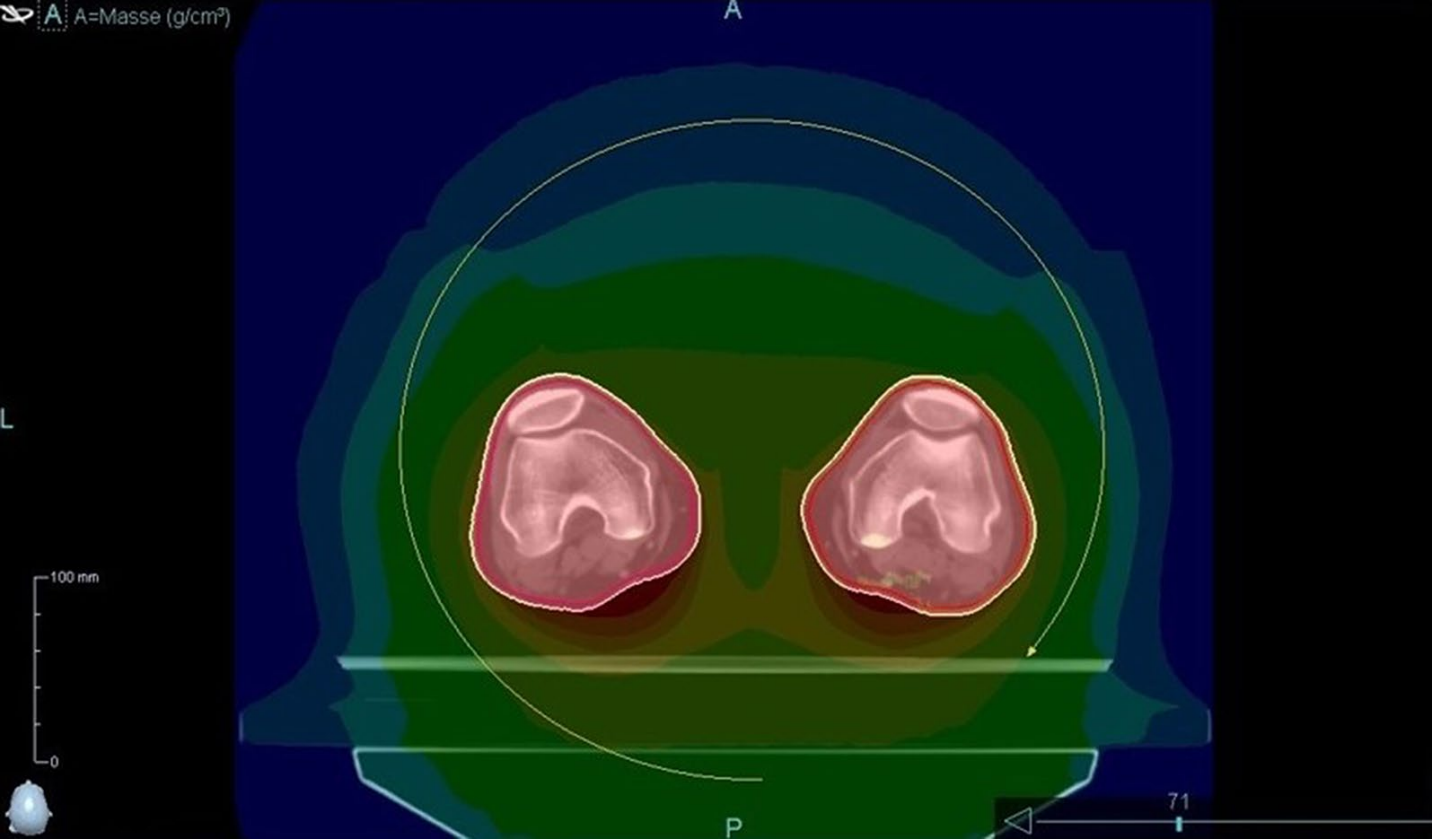
Simulation of the helical approach

**Fig. 2:**
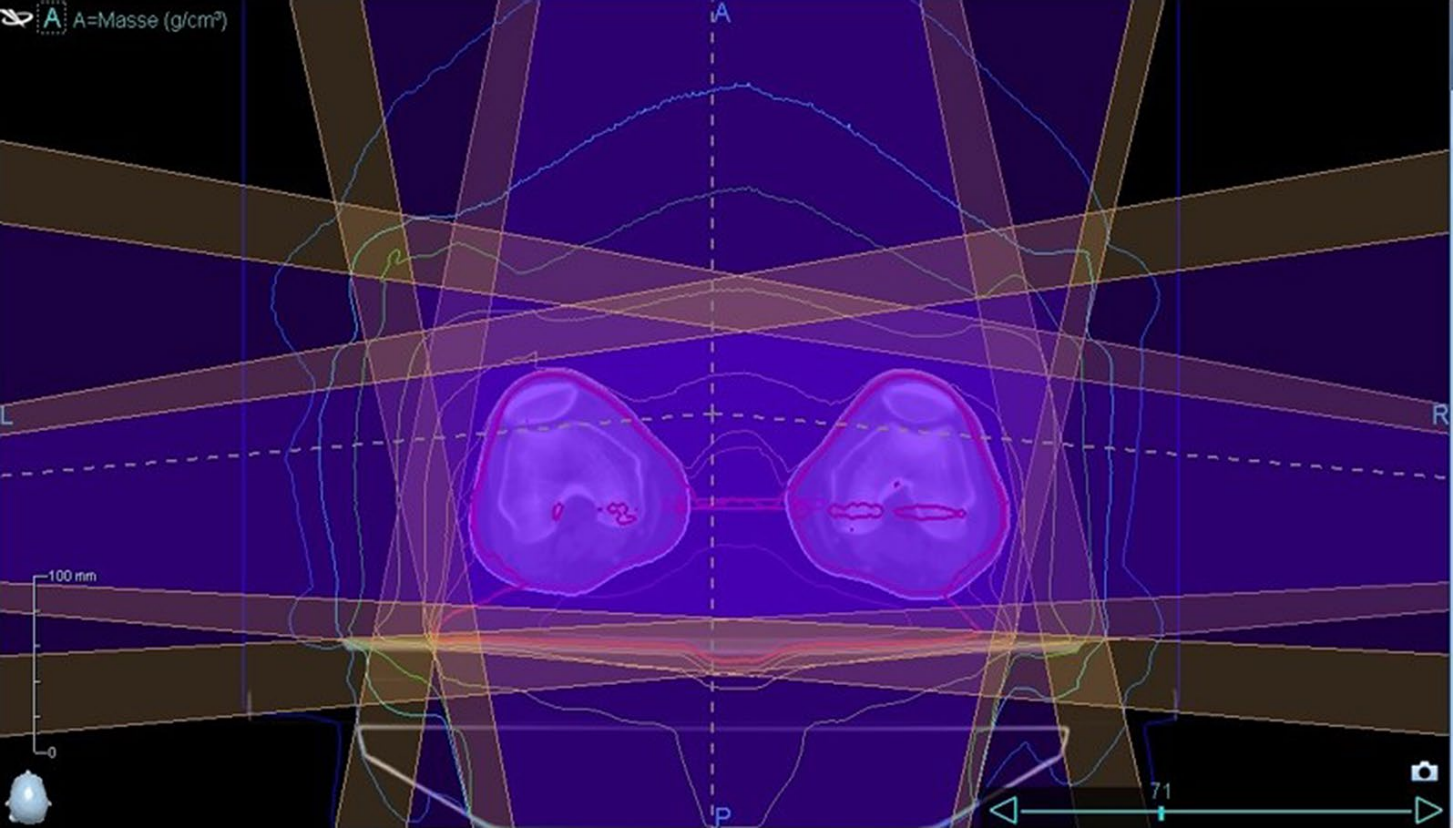
Simulation of the static four-field box approach

The matching of CT scans and all delineations and planning were performed using an Eclipse Treatment Planning unit (Varian Medical System, Palo Alto, CA, USA). To calculate the optimal dose for irradiating the junctional area, two treatment plans were fused and matched, with JT_5_ being the lowest part of the upper treatment plan and JT_6_ being the highest part of the lower treatment plan. The connecting area (JT_4–7_) was planned with 50% of the prescribed dose. Contouring of the whole body and organs at risk as well as generation of the planning target volume (PTV), sparing the lungs, were performed according to current institutional and international standards [18]. Nine patients underwent treatment planning and delivery using a helical approach (Fig. 1), and 10 patients underwent treatment planning and delivery using a static approach (Fig. 2). Both approaches used fixed jaws, a field width of 5 cm, a pitch of 0.4, and a constant feed rate fitting the pitch and prescribed dose. The modulation factors were 1.6 for the static approach and 2 for the helical approach. With the static approach, the dose was delivered from four angles, all covering the entire PTV. Treatment and planning times were equivalent between the two approaches.

**Fig. 3:**
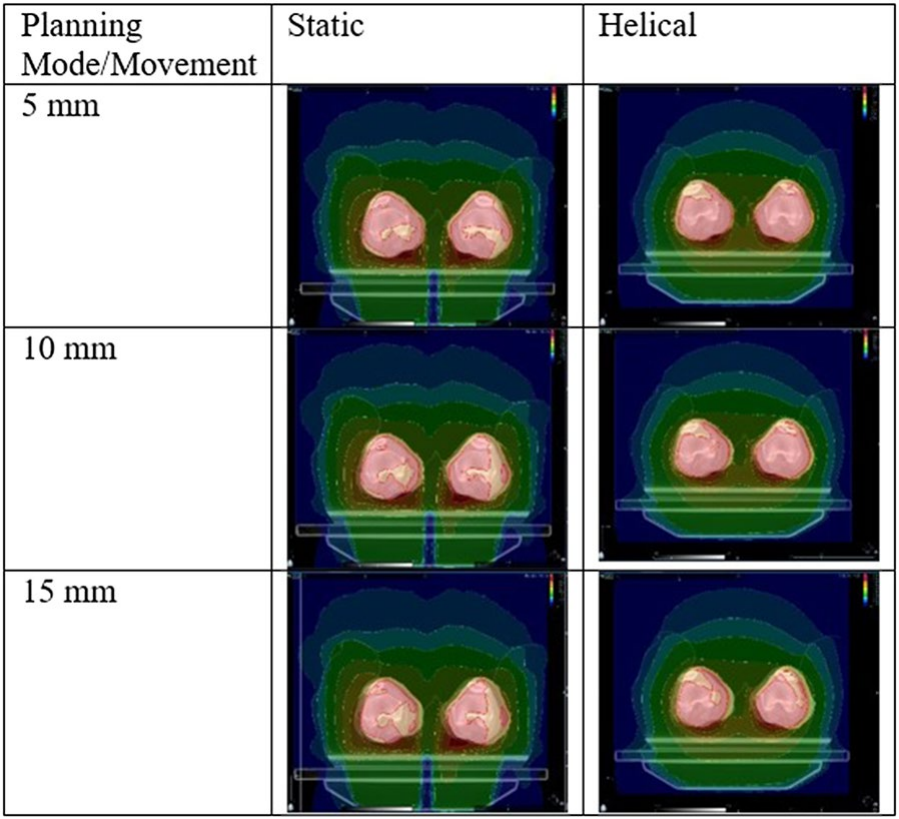
Comparison of 5-, 10-, and 15-mm lateral movement between helical and static approaches

Considering the inverse square law, which states that the dose is inversely related to the square of the distance from the radiation source, lateral movement of the patient can be compensated due to an increase in field width, which can be addressed by opening five additional leaves of the multileaf collimator. As these five additionally opened leaves do not target the PTV directly, their dose is calculated using the mean opening time of the three outermost leaves that target the PTV directly and using that calculated amount as the dose for the additionally opened leaves. This is not possible when using a helical treatment plan. Because of these technical limitations when planning TBI with the TomoTherapy Hi-ART II, the static approach offers 30 mm safety before possible subtherapeutic doses, when the position of the patient changes laterally. Because a patient must be precisely positioned during irradiation to ensure an optimal treatment outcome, a simulation demonstrates the impact of lateral movement of the patients’ legs in the static approach as compared with the helical approach (Fig. 3). In this stimulation, we virtually misplaced the patient laterally at different distances and compared the resulting changes in the DVH between the helical and static approaches.

**Fig. 4:**
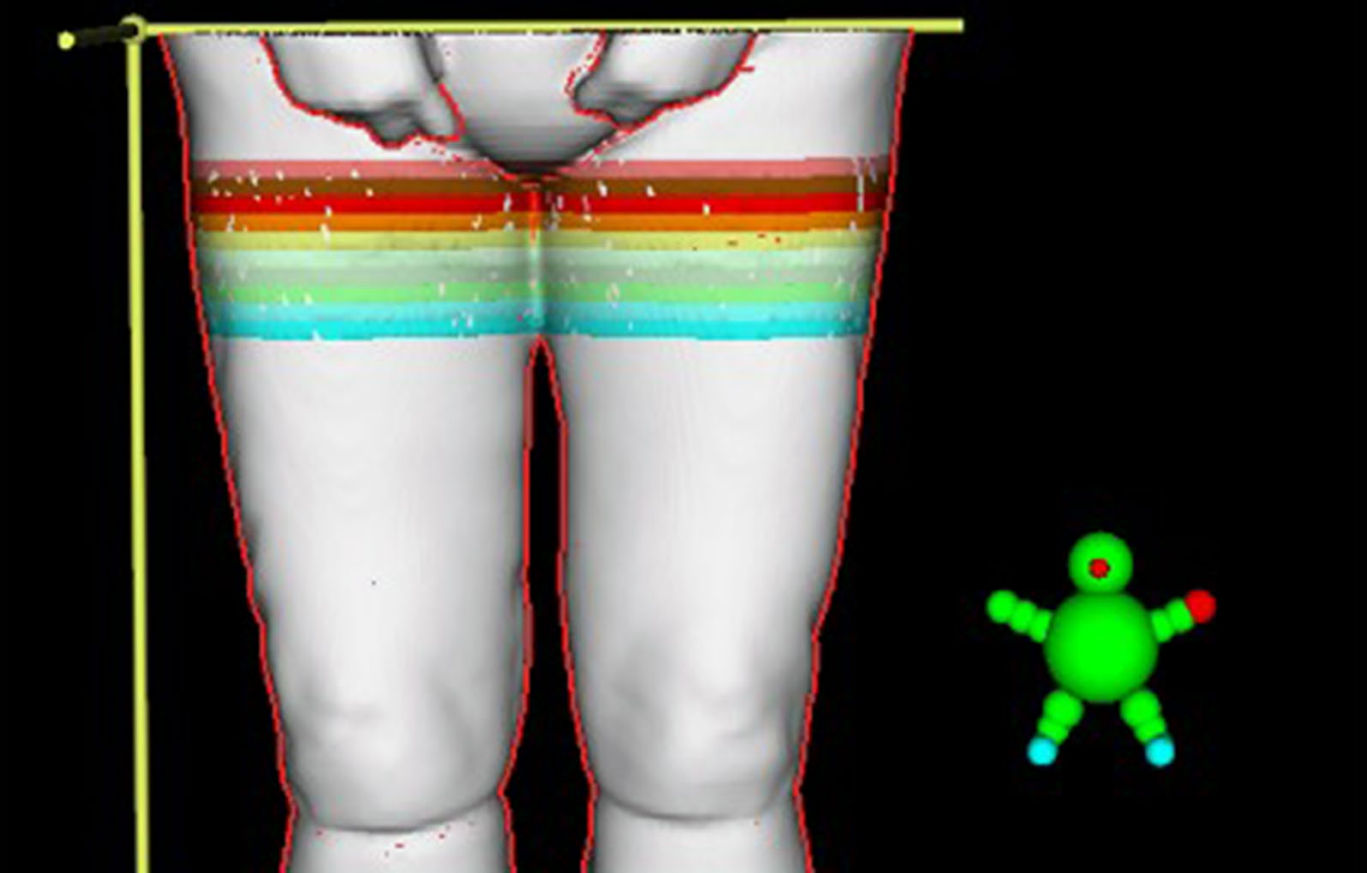
Contouring of 1 cm JT volumes (J_1_–J_10_) for patient treatment planning

To evaluate the performance of the static approach using a four-field box method compared with the usual helical approach and in consideration of TBI guidelines, we divided the JT volume between the upper and lower CT scans into ten 1-cm-thick volumes (JT_1_-JT_10_) covering the entire PTV, spanning from 5 cm above to 5 cm below the marker on the patient’s thigh (Fig. 4). This additional contouring was performed after the completion of treatment for all patients. The dose-volume histogram and the D_5_, D_50_, D_95_, D_98_, and D_mean_ as well as the homogeneity index (HI) of each JT and all ten JT volumes combined (JT_total_) were calculated. The HI was calculated using the formula proposed by Kataria et al. (HI = D_5_/D_95_) [19].

Two-sided *t* tests were used to compare groups using SPSS v26.0 (IBM, Armonk, New York, USA).

A total of 19 patients were included; 10 patients underwent a static approach to planning and treatment, and 9 patients underwent a helical approach to planning and treatment. All patients underwent TBI in preparation for BMT. The most common disease for patients in the helical group was AML, and the most common disease for patients in the static group was ALL (Table 1). In addition, two patients in the static group were treated for diffuse large B cell lymphoma and mixed phenotype acute leukaemia, respectively. The delivered dose ranged from a single 2 Gy fraction to 2 × 2 Gy, 4 × 2 Gy, or 6 × 2 Gy, resulting in a total dose of 2, 4, 8, or 12 Gy, respectively. Most patients in the helical group received 4 Gy, and most patients in the static group received 8 Gy (Table 2).

**Table 1:** Diagnosis of patients

Group	Diagnosis	*n*	%
Helical	AML	7	77.8
	ALL	2	22.2
Static	AML	2	20.0
	ALL	6	60.0
	Other	2	20.0

**Table 2:** Prescribed total dose

Group	Dose	*n*	%
Helical	2.00	2	22.2
	4.00	3	33.3
	8.00	2	22.2
	12.00	2	22.2
Static	2.00	2	20.0
	8.00	5	50.0
	12.00	3	30.0

## Results

Several dosimetric parameters were evaluated to assess the quality of radiation therapy delivered using helical versus static approaches. D_5_ of JT_total_ was calculated to assess the maximum dose absorbed by the PTV, D_95_ and D_98_ were calculated to assess the minimum dose, and D_50_ was calculated to assess the median dose as recommended by the ICRU [17] (Table 3).

**Table 3:** Dosimetric parameters for JT_total_

Group	D_5_	D_50_	D_mean_	D_95_	D_98_	HI
Helical						
Mean	118.1111	108.1221	108.3583	98.4444	94.5556	1.2001
Median	120.0000	108.6250	109.4750	100.0000	97.0000	1.1845
SD	5.78132	2.22531	3.09958	4.18662	4.97773	0.0408
Static						
Mean	116.6500	107.8050	107.2796	96.2000	93.3000	1.2125
Median	114.2500	106.0813	104.6625	95.0000	92.0000	1.2104
SD	7.37507	4.59742	4.92328	4.58984	3.94546	0.0467

For more in-depth evaluation, dosimetric parameters for each smaller fraction of the JT_total_ were also evaluated. The standard deviation (SD) of D_95_ ranged from 1.45% (JT_1_) to 7.32% (JT_6_) for the helical group and from 1.86% (JT_1_) to 9.81% (JT_5_) for the static group (Table 4). The SD of D_98_ ranged from 3.18% (JT_3_) to 7.89% (JT_7_) for the helical group and from 2.02% (JT_1_) to 8.37% (JT_5_) for the static group (Table 5). For the helical group, the highest mean D_95_ and D_98_ were in JT_9_ and JT_8_, respectively, and the lowest mean D_95_ and D_98_ were both in JT_6_. For the static group, the highest mean D_95_ and D_98_ were both in JT_2_, and the lowest mean D_95_ and D_98_ were both in JT_7_.

**Table 4:** D_95_ values of 1-cm-wide JT volumes (JT_1_–JT_10_)

Group	Min	Max	Mean	SD
Helical				
D_95_ J_1_	100.00	104.00	102.1111	1.45297
D_95_ J_2_	99.00	105.00	102.8889	2.08833
D_95_ J_3_	96.00	105.00	101.4444	2.96273
D_95_ J_4_	88.00	104.00	97.8889	6.25389
D_95_ J_5_	85.00	106.00	97.8889	7.30487
D_95_ J_6_	86.00	105.00	96.8889	7.32196
D_95_ J_7_	87.00	109.00	99.2222	7.25909
D_95_ J_8_	97.00	108.00	102.6667	4.30116
D_95_ J_9_	98.00	108.00	103.2222	3.70060
D_95_ J_10_	97.00	107.00	101.8889	3.44400
Static				
D_95_ J_1_	104.00	109.00	105.9500	1.86264
D_95_ J_2_	102.00	112.00	106.8000	3.23351
D_95_ J_3_	95.00	121.00	104.2000	8.20298
D_95_ J_4_	91.00	121.00	100.3500	9.17742
D_95_ J_5_	89.00	119.00	99.4000	9.80873
D_95_ J_6_	86.00	113.00	96.6500	8.74341
D_95_ J_7_	84.00	108.00	95.1000	8.38583
D_95_ J_8_	91.00	102.00	96.7000	4.00139
D_95_ J_9_	94.00	103.00	98.9000	2.76687
D_95_ J_10_	95.00	103.00	99.5000	2.17307

**Table 5:** D_98_ values of 1-cm-wide JT volumes (JT_1_–JT_10_)

Group	Min	Max	Mean	SD
Helical				
D_98_ J_1_	93.00	103.00	99.0000	3.67423
D_98_ J_2_	94.00	104.00	99.7778	3.86580
D_98_ J_3_	93.00	103.00	98.1111	3.17980
D_98_ J_4_	83.00	100.00	93.8889	5.92546
D_98_ J_5_	79.00	101.00	93.4444	7.28202
D_98_ J_6_	80.00	102.00	92.5556	7.51850
D_98_ J_7_	82.00	106.00	96.3333	7.88987
D_98_ J_8_	93.00	105.00	100.8889	4.34294
D_98_ J_9_	92.00	107.00	100.4444	4.66667
D_98_ J_10_	92.00	106.00	100.1111	4.22624
Static				
D_98_ J_1_	102.00	108.00	104.1000	2.02485
D_98_ J_2_	100.00	110.00	104.8000	3.08401
D_98_ J_3_	91.00	115.00	101.2000	7.68548
D_98_ J_4_	88.00	113.00	97.0000	7.90218
D_98_ J_5_	82.00	109.00	95.6000	8.36926
D_98_ J_6_	82.00	102.00	92.0000	6.39444
D_98_ J_7_	77.00	103.00	90.5000	6.98013
D_98_ J_8_	84.00	100.00	92.8000	4.84883
D_98_ J_9_	87.00	99.00	94.5000	3.83695
D_98_ J_10_	88.00	100.00	95.4000	3.68782

Mean D_50_ ranged from 106.86% in JT_1_ to 111.40% in JT_7_ for the helical group and from 104.00% in JT_10_ to 111.63% in JT_3_ for the static group. Overall, these mean doses were slightly higher than the prescribed doses (Table 6).

**Table 6:** D_50_ values of 1-cm-wide JT volumes (JT_1_–JT_10_)

Group	Min	Max	Mean	SD
Helical				
D_50_ J_1_	105.75	108.86	106.8648	1.11833
D_50_ J_2_	106.07	109.78	108.0973	1.38804
D_50_ J_3_	105.00	112.81	108.8360	2.57687
D_50_ J_4_	101.03	116.14	109.2350	5.89845
D_50_ J_5_	100.16	118.70	109.6414	7.11786
D_50_ J_6_	100.04	117.55	109.7359	7.27310
D_50_ J_7_	101.63	117.88	111.3968	6.29780
D_50_ J_8_	105.38	117.13	111.0139	4.66671
D_50_ J_9_	102.15	115.90	108.4404	4.11942
D_50_ J_10_	102.15	114.05	107.0486	3.68669
Static				
D_50_ J_1_	106.35	113.19	109.6855	2.37374
D_50_ J_2_	107.23	120.27	111.5649	4.18033
D_50_ J_3_	105.79	128.74	111.6258	7.55920
D_50_ J_4_	101.49	130.10	109.6096	9.57511
D_50_ J_5_	101.44	129.74	108.6903	9.82902
D_50_ J_6_	98.36	127.52	107.7608	9.72856
D_50_ J_7_	94.35	116.91	104.0191	8.44079
D_50_ J_8_	96.77	111.70	105.3209	4.87072
D_50_ J_9_	99.82	109.48	104.0762	2.94174
D_50_ J_10_	101.53	108.22	103.9950	2.40950

The mean HI ranged from 1.08 in JT_1_ to 1.22 in JT_6_ for the helical group and from 1.06 in JT_1_ to 1.19 in JT_6_ for the static group (Table 7). The lowest and highest HI ranges were 0.04 in JT_1_ and 0.27 in JT_4_ for the helical group and 0.06 in JT_1_ and 0.20 in JT_7_ for the static group, respectively.

**Table 7:** HI of the 1-cm-wide JT volumes (JT_1_–JT_10_)

Group	Range	Min	Max	Mean	SD
Helical					
JT_1_	0.04	1.06	1.11	1.0817	0.01418
JT_2_	0.08	1.05	1.14	1.0922	0.02963
JT_3_	0.12	1.06	1.19	1.1367	0.03876
JT_4_	0.27	1.06	1.33	1.2002	0.07262
JT_5_	0.22	1.07	1.28	1.1923	0.05726
JT_6_	0.20	1.15	1.35	1.2230	0.05548
JT_7_	0.17	1.12	1.30	1.1960	0.05903
JT_8_	0.20	1.08	1.28	1.1407	0.06622
JT_9_	0.20	1.06	1.26	1.1008	0.06224
JT_10_	0.23	1.04	1.27	1.0863	0.07052
Static					
JT_1_	0.06	1.04	1.10	1.0645	0.01813
JT_2_	0.10	1.06	1.16	1.0838	0.02973
JT_3_	0.12	1.08	1.20	1.1248	0.03682
JT_4_	0.11	1.11	1.23	1.1639	0.04496
JT_5_	0.16	1.12	1.28	1.1721	0.04711
JT_6_	0.14	1.14	1.28	1.1892	0.05285
JT_7_	0.20	1.12	1.32	1.1732	0.06156
JT_8_	0.08	1.09	1.16	1.1334	0.02473
JT_9_	0.07	1.07	1.13	1.0958	0.02411
JT_10_	0.09	1.04	1.13	1.0676	0.02738

No significant differences in any dosimetric parameter were found between helical and static groups (Table 8).

**Table 8:** Statistical comparisons of JT_total_ dosimetric parameters between helical and static groups

Parameter	*t*	*p*	Mean difference	Standard error of the difference
HI	−0.614	0.548	−0.01241	0.02022
D_98_	0.613	0.548	1.25556	2.04973
D_95_	1.109	0.283	2.24444	2.02382
D_mean_	0.564	0.580	1.07868	1.91403
D_50_	0.188	0.853	0.31715	1.68945
D_05_	0.477	0.640	1.46111	3.06587

## Discussion

TBI is commonly used in allogeneic BMT conditioning regimens [20]. Patients often receive parallel chemotherapy to eradicate any malignant cells from the patient’s body, especially in the blood and haematopoietic tissue, such as bone marrow. In addition, in preparing for BMT, the patient’s own immune system is targeted to later implement the allogeneic transplant [21]. These systemic treatments result in high levels of toxicity. However, TBI is a feasible treatment option that can reduce total levels of toxicity [22]. In haematological malignancies, it is important to maximize treatment success while minimizing side effects. Thus, in this study, we aimed to maximize dose homogeneity by optimizing JT treatment and creating a robust plan for patients taller than 135 cm, who require two treatment plans when using the TomoTherapy® Hi-ART II.

Regarding D_50_ values, we observed slightly higher treatment doses than prescribed doses for both the helical and static groups. Although this may be acceptable, because there are no organs at risk in the junctional area that might experience more toxicity from an increased radiation dose [23], this discrepancy is still important and should be addressed during treatment planning.

Dosimetric parameters for JT_total_ showed only a narrow margin between median and mean values, indicating the existence of few outliers and a symmetric distribution suggestive of good dose distribution. Upon further analysis of 1-cm-wide volumes, the highest SD in D_95_ and D_98_ were observed in JT_5_, JT_6_, and JT_7_. These junctional volumes represent the last region of the upper treatment plan (J_5_) and the first two regions of the lower treatment plan (JT_6_ and JT_7_). Although overall coverage was acceptable, even in these specific junctional volumes, additional research may be needed to achieve ideal dosimetric parameters for this 3-cm region when planning TBI.

On the other hand, the mean value of D_95_ for JT_1_–JT_10_ ranged from 96.89% to 103.22% for helical group and 95.10–106.80% for the static group, while the mean value of D_98_ was only slightly lower, ranging from 92.56% to 100.89% for the helical group and 90.50% and 104.80% for the static group. Thus, the minimum dose did not fall rapidly between the dose delivered to 95% of the PTV and the dose delivered to 98% of the PTV, suggesting good overall dose coverage and dose-volume histograms for both helical and static groups.

The HI is commonly used for quality assessment in TBI [17, 19], with the Radiation Therapy Oncology Group (RTOG) recommending a maximum HI < 2 [17]. The 19 patients included in this study received treatment with a mean HI for the JT_total_ of 1.20 in the helical group and 1.21 in the static group. Considering individual HI values for JT_1_–JT_10_, mean HI ranged from 1.08 to 1.22 in the helical group and 1.06 to 1.19 in the static group. These values are well under the maximum HI recommend by the RTOG. Even the maximum HI, found in JT_6–7_ with values of 1.35 in the helical group and 1.32 in the static group were still well below the limit of a minor violation, defined as a value between 2 and 2.5. Therefore, both helical and static approaches to delivering treatment showed acceptable homogeneity. The conformity index was not calculated, because the PTV was equal to the targeted volume [24].

Furthermore, statistical analysis showed no significant differences between helical and static groups considering any dosimetric parameter, indicating comparable performance between both treatment planning and delivery approaches.

Importantly, however, using a four-field box to irradiate the JT volume in the static approach provides 30 mm additional safety in case the patient changes their position. Technical limitations of the TomoTherapy^®^ Hi-ART II do not allow the same possibility when using a helical approach. Therefore, considering the otherwise equivalent performance between static and helical approaches, using a four-field box for patients taller than 135 cm undergoing TBI may be considered and preferably chosen over the helical approach. Previous studies show that a virtual bolus can be used to reduce HT setup error when using a helical treatment regimen for the legs and JT [24, 25]. However, use of a virtual bolus can also lead to underdosage, especially in the smaller parts of the legs, as well as overdosage. In this regard, the inverse square law gives the static approach an advantage, as it prevents underdosage and overdosage as long as the setup error does not exceed the additional safety given by opening five additional leaves [26].

## Conclusions

We compared the performance of helical versus static approaches to planning and delivering radiation to the JT volume in patients taller than 135 cm, who required two planning CT scans before undergoing TBI in allogeneic BMT conditioning regimens. The static approach utilized a four-field box to deliver the prescribed dose. Based on our institution’s experience, both helical and static treatment plans meet the ICRU and RTOG guidelines, and the static planning method is not inferior to the commonly used helical planning method. Furthermore, simulations and the physical law suggest that additional safety can be provided when using the static approach to treatment planning, which avoids the challenges of using a virtual bolus, making the four-field box a feasible method for TBI planning and delivery.

## Data Availability

All data relevant to this publication have been included into the manuscript’s body.

## Abbreviations

ALL: Acute lymphoblastic leukaemia
AML: Acute myeloblastic leukaemia
BMT: Bone-marrow transplantation
CT: Computed tomography
HI: Homogeneity index
ICRU: International Commission on Radiation Units and Measurements
JT: Junctional target
PTV: Planning target volume
RTOG: Radiation Therapy Oncology Group
TBI: Total body irradiation
